# Job demands and resources as drivers of exhaustion and leaving intentions: a prospective analysis with geriatric nurses

**DOI:** 10.1186/s12877-023-03829-x

**Published:** 2023-03-23

**Authors:** Marlen Rahnfeld, Johannes Wendsche, Jürgen Wegge

**Affiliations:** 1 Faculty of Psychology, TU Dresden, Zellescher Weg 17, D-01069 Dresden, Germany; 2 Federal Institute for Occupational Safety and Health, Fabricestraße 8, D-01099 Dresden, Germany

**Keywords:** Exhaustion, Health, Job demands, Resources, Person-organisation fit, Leaving intentions

## Abstract

**Background:**

Nurses show a high prevalence of exhaustion and increased leaving intentions. With this study, we integrate established research about turnover intention with recent burnout literature and present a theoretical model that combines both. The aim of this study was to examine job demands (time pressure, social conflicts) and resources (job control, supervisor support, task identity, person-organisation fit) as drivers and health and age as moderators for the relationships between exhaustion and nurses’ organisational and professional leaving intentions.

**Methods:**

We analysed data from a standardised paper-pencil questionnaire survey with a prospective, two-wave (12 months apart) study design. In total, 584 nurses participated at Time 1 (t1). The final sample at Time 2 (t2) was *n* = 222 nurses (38%; age: *M* = 41.1 years, *SD* = 11.0; 88% females).

**Results:**

We identified time pressure as job demand and job control, task identity, and person-organisation fit as resources that drive the relationships of exhaustion (mean between both times of measures) and organisational and professional leaving intentions. The relationships to organisational leaving intentions decreased with nurses’ age and the relationships to professional leaving intentions increased for nurses who had poorer self-rated health. We found indirect effects of exhaustion for relationships between job demands and nurses’ leaving intentions. Relationships to exhaustion remained significant after adjusting for depressive mood.

**Conclusion:**

Insights from this study can be used both by employers and employees. Redesigning work might be a promising approach to improve nurses’ well-being and retention in this profession. Geriatric care facilities should include the concept of person-organisation fit into their personnel selection process.

Europe’s population is ageing [1]. This not only means that more people need care as they age, but also implies that the workforce is shrinking as it ages. This includes the people who care for older people: nurses [2]. Furthermore, the nursing profession is challenged by adverse working conditions such as significant workloads, emotional demands, and little job control, staff shortage, a poor reputation in society, low wages, and unfavourable career aspects [3–5]. Not unexpectedly, this occupational group has high rates of the burnout syndrome, which often means long absences from work [6]. For many years, absence and turnover rates as well as the number of nurses leaving the profession prematurely have been far above average [4, 7]. Since early retirees are no longer available to the health and geriatric care sector, nurses must be a priority—whether that be in winning new nurses to the profession, preserving the health and work abilities of current nurses, or preventing future premature exits from the profession.

With this study, we integrate established research about turnover intention with recent burnout literature and present a theoretical model that combines both. In doing so, we look at both well-known and rather unexplored antecedents of exhaustion and leaving intentions. Next, both age and health are central in times of demographic change but have received little attention in theoretical models. This study therefore extends current insights by investigating the moderating effects of age and health in the prediction of nurses’ leaving intentions through a prospective design. A prospective view on relationships credits the developmental character of burnout symptomatology [8] and leaving intentions [4]. Not least, this study clarifies different mechanisms regarding whether a nurse wants to leave the organisation or the profession. From a practical perspective, knowing more about the risks and benefits that lie in the person, in the work and social environment, or in both may enable organisations in health and geriatric care to build healthier workplaces to retain nurses.

## Theoretical background

To master the challenges of demographic change and a changing world of work, these issues must be reflected upon in theory and models. For this, an adequate model uniting turnover and burnout is needed. Although there is a body of research [9–13], there is to date no generally accepted theoretical model for explaining nursing turnover [14]. A theoretical model that has been widely established in work and organisational psychology and has been used in nursing research [15, 16] is the job demands-resources (JD-R) model, developed by Bakker and Demerouti [17, 18]. Generally, the JD-R model describes health and motivational processes at work, which predict several outcomes. Introduced in 2001, the model has been used to explain job performance or job satisfaction; additionally, it has been widely used in studies with nurses. Yet, it probably earned the most attention for its explanation of the burnout syndrome, with an important and much-cited article in this area by Bakker, Demerouti, and Verbeke, which was published in 2004 [19]. Accordingly, the concurrence of high demands and low resources is likely to lead to burnout [19, 20]. The JD-R model’s assumptions and results regarding burnout, performance, and even turnover intentions, in other professions in addition to nursing, form an adequate basis to illustrate the complex interplay of multiple variables relating to the socio-technical work system and for explaining mechanisms that precede burnout and turnover intentions as well as identifying beneficial variables for maintaining health in the nursing profession. Although the JD-R model works well to explain central work processes and has demonstrated its validity in numerous studies, some open questions remain when explaining nurses’ leaving intentions, which require additions for the special application in the care context.

## Study aims

**Fig. 1:**
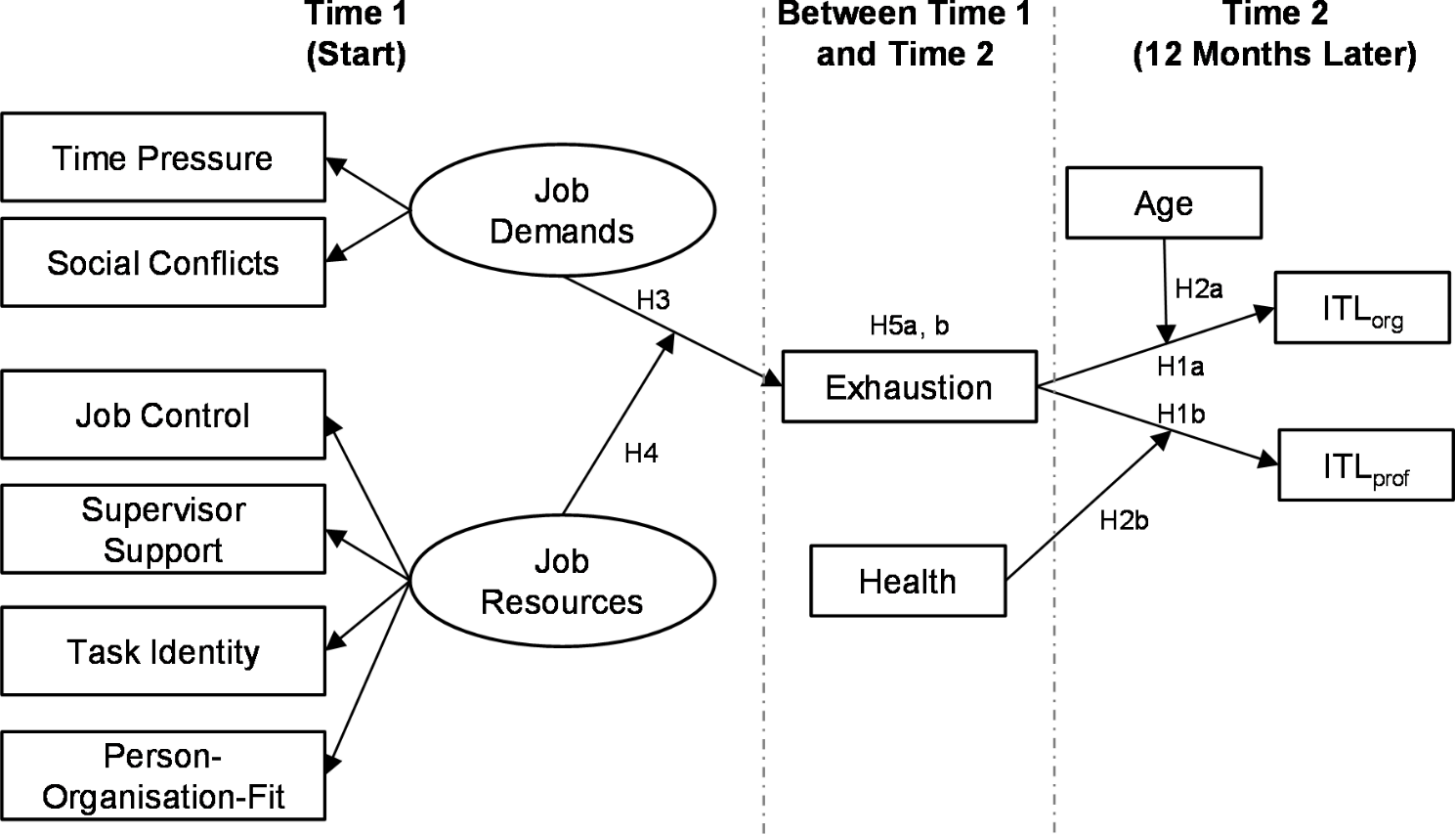
Conceptual Research Model. Note: ITL_org_ = intention to leave the organisation, ITL_prof_ = intention to leave the profession

The aim of the present, prospective study is to investigate the relationships between job demands (such as time pressure and social conflicts) and job or personal resources (such as task identity, job control, supervisor support, and person-organisation fit [PO fit]), and exhaustion as drivers of organisational and professional leaving intentions in a sample of geriatric care nurses who were studied over the course of 12 months. The NEXT study reveals that approximately 80% of those who left the profession had begun to consider the move seriously within the previous 12 months [4]. Specifically, this study extends prior research by regarding age and health as moderators in the relationship between exhaustion and leaving intentions. According to the research discussed in the following paragraphs, we tested the several hypotheses as part of a comprehensive research model (see Fig. 1).

**Job Demands and Resources as Predictors of Exhaustion.** Numerous studies, which have been summarised in several literature reviews [9–11] and meta-analyses [12, 13], have presented a wide range of antecedents of leaving intentions and behaviour regarding decisions to change jobs, organisations, or professions. Additionally, several moderating and mediating variables that influence and underscore the processes have been identified, such as effects of strain (e.g., burnout) and attitudinal variables (e.g., job satisfaction and organisational commitment) that are associated with work (e.g., job demands and job resources), organisational (e.g., leadership), and individual variables (e.g., age and gender) [14, 21]. Thus, prior research has revealed that leaving intentions rarely emerge quickly. Rather, this decision seems to be a long-term and stepwise process that is influenced by several moderators and mediators [12, 22].

There is substantial empirical evidence for many demands and resources, which demonstrates the model’s validity in nursing [15]. Time pressure and conflicts with co-workers, both considered to be job demands, are known to be antecedents of exhaustion in the nursing profession [8, 16]. However, specific demands and resources may have particular roles in predicting nursing burnout and leaving intentions but have not earned adequate attention in past research, such as task identity and PO fit. Task identity, a work characteristic proposed by Hackman and Oldham [23], is featured in other major theories in work psychology, such as the action regulation theory by Hacker [24]. Task identity provides employees with a sense of contentment, wholeness, and an improving quality of working life [25]. However, few studies with nurses have investigated its role and presented its predictive value for explaining turnover [26]. For the nursing profession, it may be relevant as an important resource and representative for a healthy work design. In 2017, Bakker and Demerouti amended the JD-R model by claiming that personal characteristics, such as optimism, can be resources and can interact with job resources. The perceived fit between a person and an organisation represents this assumption, as reflected in the concept of PO-fit [27]; however, the concept has not garnered significant attention in nursing studies or in combination with the JD-R model. Therefore, perceived PO fit is included in this study; it can be regarded as a combination of both job and personal resources. PO fit is a facet of the person-environment fit and is defined by Kristof [27] as “the compatibility between people and organisations that occurs when: (a) at least one entity provides what the other needs, or (b) they share similar fundamental characteristics, or (c) both” (p. 4–5). Several studies and meta-analyses have found direct, negative relationships of PO fit with strain outcomes such as exhaustion as well as turnover intentions [12, 28, 29]. For this study, we also used job control as task-related job resource, and supervisor support which is an important task- and emotion-related resource from the social environment. Several studies have underscored the moderating role of resources from the job and social environment as a burnout predictor [30] while others have not found clear evidence for this [19]. Therefore, our first two hypotheses are as follows.

### Hypothesis 1

Job demands are positively associated with exhaustion.

### Hypothesis 2

Resources reduce the positive relationship between job demands and exhaustion.

**Exhaustion and Leaving Intentions.** Exhaustion is one of three major symptoms of burnout syndrome, in addition to cynicism (depersonalisation) and inefficacy (reduced personal accomplishment) [8]. Two large-scale and prospective international studies regarding nursing turnover, the 2003 nurses’ early exit (NEXT) study and the 2011 registered nurse forecasting (RN4CAST) study, have revealed that burnout is an important precursor of leaving the profession [7, 31]. A meta-analysis by Alarcon [32], although not specific to nurses, presents a notable correlation of ρ = 0.39 between emotional exhaustion and turnover intentions. Similar results are yielded in the meta-analysis by Swider and Zimmermann [33] with an average correlation of 0.33 to turnover. Although proposed by many researchers—especially in the NEXT and RN4CAST studies—the majority of models do not distinguish between the intention to leave the current job or organisation and the intention to leave nursing entirely, although research suggests different antecedents for each [3, 7, 34]. Therefore, we explicitly include both in our third and fourth hypothesis.

### Hypothesis 3

Exhaustion is positively associated with the intention to leave the organisation (H3a) and the profession (H3b).

### Hypothesis 4

Exhaustion demonstrates indirect effects on the relationship between job demands and intention to leave the organisation (H4a) and the profession (H4b).

**Age and Health as Moderators.** The demographic change inevitably produces questions regarding the roles of age and health, but this is rarely reflected in turnover models. The JD-R model, for instance, offers no explicit consideration of age and health. In this research, age and health are considered as moderators in the relationship between exhaustion and the intention to leave the organisation or the profession. While the majority of studies and reviews have reported turnover to be stronger among younger nurses [4], others have found that older nurses are more likely to leave the profession [7]. How can researchers account for these inconsistencies? Again, it is important to carefully differentiate between leaving the organisation and leaving the profession [34]. Young nurses are more likely to change jobs for career-related reasons: they may have fewer family obligations and thus are more mobile, and they may face fewer responsibilities and lower status in the early stages of their career. However, older nurses’ desire to leave the profession and retire prematurely may be primarily due to health reasons. It seems plausible that poor health is generally a strong predictor of early retirement [35, 36]. In a study of nurses in Brazil, the intention to leave nursing was three times more likely when nurses reported their health as poor or very poor [37]. In a Danish cohort study, nurses reporting poor health were more likely to enter early retirement [38]. The authors of the NEXT study [4] also suspect two kinds of leavers: so-called motivated leavers on the one hand, who were mostly of younger ages. On the other hand, resigning leavers were typically in poor health and would rather strive for early retirement. Hence, age seems more relevant when changing organisations, and health seems to be particularly important in leaving the profession. However, neither the role of moderators, such as age and health, nor the differences depending on the outcome are found in the JD-R model; furthermore, these factors have not been ascertained explicitly by past research. We are unaware of any study that has explicitly tested moderating effects of age and health in the relationship between exhaustion and intention to leave. This leads us to our fifth hypothesis.

### Hypothesis 5

Age and health moderate in the relationship between exhaustion and intention to leave. Age reduces the positive relationship between exhaustion and intention to leave the organisation (H5a), and health reduces the positive relationship between exhaustion and intention to leave the profession (H5b).

## Method

### Procedure and participants

We used prospective two-wave data (t1 = start, t2 = 12 months after t1) from a German study regarding working conditions, health, and turnover of nurses in geriatric care [5, 39–45] (all previous publications based on this data set were revealed to the Editor). The German Research Foundation approved the protocol of this study. According to occupational stress and turnover research [4, 22], 12 months is adequate to detect changes in exhaustion and leaving intentions in response to work characteristics.

At the beginning of this study (t1), we identified all geriatric care services in the Federal State of Saxony (Germany) from an online database. Then, we contacted the management and administration of a random sample of 240 services (approximately 19% of the full sample) by phone or email to invite them to participate; 50 care services enrolled in the study. Thereafter, members of the research team visited the facilities and presented the study goals and procedures to the management and staff. Nursing staff from these institutions were also invited to participate in a survey, for which consent was granted by the management. Anonymity and privacy compliance were assured. Participants provided written consent to participate in the study. In total, 584 nurses completed the standardised paper-and-pencil questionnaires at work during a paid work break or at home. Twelve months later, a final sample of *n* = 222 nurses (38%; age: *M* = 41.1 years, *SD* = 11.0; females: *n* = 196, 88%) participated in the survey again, which we considered a satisfactory response rate, as the mean annual organisational turnover rate of registered nurses was approximately 17% at t1 in this sample. These nurses were employed in 44 organisations (care setting: *n*_home care_ = 18, *n*_nursing homes_ = 26; type of ownership: *n*_non-profit_ = 25, *n*_for-profit_ = 19). Care setting and ownership did not significantly correlate with the outcomes of exhaustion and intention to leave.

### Measures and data collection

We administered the measures at two time points to study prospective relationships between the variables under investigation and to reduce the impact of a possible common method bias [46]. At t1, we assessed job demands and resources as independent variables and control variables. At t1 and t2, we assessed the mediating variable of exhaustion, the moderators of subjective health and age, and depressive mood as confounder. We assessed the dependent variables of intention to leave the organisation and intention to leave the profession at t2. Descriptive statistics and measures of reliability for all variables are provided in the results section.

**Job Demands at t1**. We assessed time pressure and social conflicts as job demands. Time pressure was assessed through four items from the German questionnaire on perceived work intensity and activity latitude [47] (sample item: ‘The required pace of work is very fast’; responses range from 1 = ‘No, completely wrong’ to 4 = ‘Yes, completely true’). We assessed social conflicts via two items from the ‘Salutogenetic Subjective Work Analysis’ (SALSA; sample item: ‘There is often tension at the workplace’; responses ranged from 1 = ‘Absolutely disagree’ to 5 = ‘Absolutely agree’) [48]. We used the mean of responses as scale indicators. Higher scale values indicated higher perceived time pressure and more social conflicts.

**Resources at t1**. We assessed job control, supervisor support, task identity, and PO fit by their mean scores of respective items; higher scores indicated a greater availability of each resource. Job control was assessed as decision authority via four items from the German questionnaire on perceived work intensity and activity latitude [47] (sample item: ‘I can plan and organise my work autonomously’; see the response format above). *Supervisor support* (seven items; sample item: ‘My supervisor lets me know how well I have done my work’) and *task identity* (three items; sample item: ‘At this job I do something as a whole, in complete’) were assessed with items from the SALSA questionnaire [48] (see the response format above). We assessed perceived PO fit via four items based on Kristof [27] (sample item: ‘This organisation fits me well’; responses ranged from 1 = ‘Do not agree at all’ to 7 = ‘Fully agree’).

**Exhaustion at t1 and t2**. We assessed exhaustion at t1 and t2 via the mean score of five items from the emotional exhaustion scale of the German version [49] of the Maslach Burnout Inventory (MBI; sample item: ‘I feel burned out from my work’; responses ranged from 1 = ‘Never’ to 7 = ‘Every day’) [50]. In our model, exhaustion is a mediating variable that connects work characteristics to leaving intentions; therefore, we modelled it as the mean of t1 and t2 in our analyses by applying the midpoint estimation technique proposed by Kim and Beehr [51, 52]. This approach is warranted if variables are more state-like, such as exhaustion, and change significantly between assessments. This was revealed by results of a *t*-test for dependent samples (change between t1 and t2 *M*Δ = 0.17; *t*(221) = -2.22, *p* = .028).

**Intention to Leave (ITL) at t2.** We used items from the European NEXT study [34] to measure nurses’ leaving intentions. Nurses’ intention to leave the organisation (ITLorg; sample item: ‘How often during the past 12 months did you think about leaving your organisation?’) and nurses’ intention to leave the profession (ITLprof; sample item: ‘How often during the past 12 months did you think about leaving your profession?’) were each assessed with two items on a 5-point frequency scale (1 = ‘Never’ to 5 = ‘Daily’).

**Health and Age as Moderators.** We assessed nurses’ subjective health with the item ‘How would you describe your general state of health?’ from the German SF-12 health survey [53] on a 5-point rating scale ranging from 1 = ‘Bad’ to 5 = ‘Excellent’ at t1 and t2. Again, we used the midpoint estimation technique between the measurements. At the mean, self-reported health increased slightly within the past 12 months in this sample (change between t1 and t2 *M*Δ = -0.08; *t*(221) = 1.80, *p* = .073). Age was assessed in years at t1 and t2 (in the moderator analyses, we used the measure at t2).

**Control Variables.** In line with research regarding occupational stress and turnover [10–13], we assessed several control variables at t1 that could impact levels of exhaustion and leaving intentions. These were level of qualification (0 = ‘Nursing assistant’, 1 = ‘Registered nurse’), leadership responsibility (0 = ‘Without’, 1 = ‘With’), sex (0 = ‘Female’, 1 = ‘Male’), job contract (0 = ‘Permanent’, 1 = ‘Fixed-term’), and working hours (hours per week). One important confounder of exhaustion is depression, and recent studies have revealed strong correlations between these variables [54]. Therefore, we assessed depressive mood at t1 and t2 via the average of five reverse-coded items from the WHO-5 well-being index [55]. Research supports that the WHO-5 is valid to screen for depression [56]. Again, we used the midpoint estimation technique between these measurements to construct a variable that is parallel to exhaustion. However, in contrast to the findings for exhaustion, a significant change in depressive mood was not confirmed in this sample (change between t1 and t2 *M*Δ = -0.06; *t*(221) = 0.92, *p* = .361).

### Addressing missing data

The amount of missing data was low in this study (above 1% were: supervisor support had *n* = 3, ITLorg had *n* = 4 and ITLprof had *n* = 4). The results of Little’s test indicated that data were missing completely at random (Χ²(62) = 49.06, *p* = .883). Therefore, we used the expectation maximisation algorithm in SPSS 26 for data imputation [57].

### Hierarchical data structure

As our data were nested within *n* = 44 higher organisational units (i.e., care services), we calculated intra-class correlations (ICC1) to verify whether hierarchical data analysis would be necessary. However, the variance that would potentially be explained by organisational membership was rather low for our central outcome of exhaustion (ICC1 < 0.001), ITLorg (ICC1 = 0.045), and ITLprof (ICC1 < 0.001). As the ICCs in our study were lower than in other multilevel studies from organisational research [58] (range from 0.15 to 0.30), we decided that analyses on the individual level were warranted.

### Data analyses

In the first step, we tested the measurement model and examined the distinctiveness of our core constructs—job demands, resources, exhaustion, and intention to leave—by conducting a series of confirmatory factor analyses (CFAs) with AMOS 26. We compared the models based on results regarding a X²-test (*p* > .05), the root mean square error of approximation (RMSEA < 0.06), the comparative fit index (CFI > 0.90), and the Akaike information criterion (AIC; lower values indicate a better fit) according to cut-offs as outlined by Hu and Bentler [59] and Kline [60].

Second, we calculated descriptive statistics and intercorrelations between all variables with SPSS Version 26. We identified relevant confounders for our main independent variables: exhaustion and leaving intentions. Third, we used hierarchical regression analyses to test our hypotheses. To examine moderating effects, we calculated product-interactions terms of the *z*-standardised independent and moderating variables [61]. In turn, we examined patterns of significant interaction effects by plotting the relationships between the independent and dependent variables at the moderating variables’ level (± 1 *SD*) and further by conducting simple slope analyses [61].

Finally, indirect effects of exhaustion were probed using the SPSS-PROCESS-plugin from Hayes [62] (5,000 bias-corrected bootstrap samples).

We applied two-tailed statistical tests for all analyses with a significance level of at least *p* < .05 (to confirm indirect effects, the 95% confidence interval should exclude zero).

## Results

### Preliminary analyses

#### Construct validity

To test the validity of our measurements, we conducted CFAs. Regarding the distinctiveness of the six independent variables—time pressure, social conflicts, job control, supervisory support, task identity, and PO fit—we found that the fit of a six-factor-model (Χ²(237) = 437.76, *p* < .001, RMSEA = 0.062, CFI = 0.903, AIC = 611.76) was superior to a one-factor-model with all variables loading on one factor (Χ²(252) = 1328.15, *p* < .001, RMSEA = 0.139, CFI = 0.479, AIC = 1472.15) and a two-factor model with each variable loading on a corresponding latent job demands factor and a resources factor (Χ²(251) = 1079.03, *p* < .001, RMSEA = 0.122, CFI = 0.599, AIC = 1225.03).

For the dependent variables of leaving intentions, we found that a two-factor-model with items loading on the factors ITLorg and ITLprof (Χ²(1) = 0.148, *p* = .701, RMSEA < 0.001, CFI = 1.00, AIC = 26.15) fit our data better than a one-factor-model with all items loading on a general factor of leaving intentions (Χ²(2) = 101.39, *p* < .001, RMSEA = 0.474, CFI = 0.606, AIC = 126.39). We further found that a three-factor-model with exhaustion, ITLorg and ITLprof at t2 (Χ²(24) = 51.87, *p* = .001, RMSEA = 0.072, CFI = 0.971, AIC = 111.87) was superior to two two-factor-models with items of exhaustion loading with items of ITLorg (Χ²(26) = 144.14, *p* < .001, RMSEA = 0.143, CFI = 0.878, AIC = 200.14) or ITLprof (Χ²(26) = 111.83, *p* < .001, RMSEA = 0.122, CFI = 0.911, AIC = 167.83) on one common factor. Moreover, a two-factor-model of exhaustion and depressive mood at t2 (Χ²(34) = 111.80, *p* < .001, RMSEA = 0.102, CFI = 0.943, AIC = 173.80) was superior to a one-factor-model (Χ²(35) = 413.41, *p* < .001, RMSEA = 0.221, CFI = 0.723, AIC = 473.41). According to these results, we considered our measures to be distinct constructs.

#### Method variance

As we assessed all variables with the questionnaire method, we conducted a further CFA, which suggested that the common method factor variance [46] of our variables was 1%. Therefore, regarding the prospective design of our study and this low value, the chance that a common method factor bias might have affected our results is low.

#### Descriptive statistics, ICC, and Cronbach’s alphas

In Table 1, we present the descriptive statistics, intercorrelations, and Cronbach’s alphas as measurements of reliability for all study variables. Regarding the potential confounders, we found that only the depressive mood between t1 and t2 substantially (*r* > .20) related to exhaustion between t1 and t2 (*r* = .62) and to ITLorg (*r* = .31) and ITLprof (*r* = .39) at t2. The relationships between ITLorg at t2 and qualification (*r* = .14) as well as age (*r* = .15) at t1 were significant but small. Therefore, we adjusted only for depressive mood in our analyses, to test a more parsimonious model and to prevent overfitting in regression analyses.

**Table 1:** Descriptive Statistics and Intercorrelations of Study Variables

		1	2	3	4	5	6	7	8	9	10	11	12	13	14	15	16	17	18	19	20	21	22	23
1	Qualification	-																						
2	Leadership Position	0.24**	-																					
3	Age	0.10	0.22**	-																				
4	Sex	0.08	− 0.01	− 0.19**	-																			
5	Contract	− 0.09	− 0.19**	− 0.21**	0.09	-																		
6	Working Hours	0.10	0.24**	− 0.08	0.11	− 0.04	-																	
7	Depressive Mood (t1)	0.06	− 0.06	0.03	0.07	− 0.07	− 0.02	(0.83)																
8	Depressive Mood (t2)	0.10	− 0.09	− 0.02	0.02	0.02	0.02	0.46**	(0.89)															
9	Depressive Mood (t12)	0.09	− 0.09	0.01	0.06	− 0.03	< 0.01	0.84**	0.87**	(0.87)														
10	Health (t1)	0.06	0.06	− 0.18**	0.15*	0.01	0.08	− 0.22**	− 0.50**	− 0.42**	-													
11	Health (t2)	− 0.05	0.01	− 0.25**	0.19**	0.14*	0.13*	− 0.30**	− 0.25**	− 0.32**	0.48**	-												
12	Health (t12)	< 0.01	0.04	− 0.25**	0.19**	0.08	0.12	− 0.30**	− 0.44**	− 0.44**	0.87**	0.85**	(0.64)											
13	Time Pressure (t1)	0.11	0.02	0.14*	− 0.06	− 0.02	0.02	0.22**	0.18**	0.23**	− 0.13	− 0.15*	− 0.16*	(0.81)										
14	Social Conflicts (t1)	0.06	0.08	− 0.10	0.11	< 0.01	0.19**	0.25**	0.23**	0.29**	− 0.19**	− 0.11	− 0.18**	0.22**	(0.68)									
15	Job Control (t1)	0.21**	0.43**	− 0.01	0.03	− 0.18**	0.22**	− 0.16*	− 0.16*	− 0.18**	0.13*	0.18**	0.18**	0.03	− 0.03	(0.75)								
16	Task Identity (t1)	− 0.09	− 0.09	0.09	− 0.12	− 0.03	− 0.16*	− 0.33**	− 0.17**	− 0.29**	0.11	0.16*	0.15*	− 0.17*	− 0.28**	0.10	(0.66)							
17	Supervisor Support (t1)	0.01	− 0.11	− 0.09	0.04	0.06	0.01	− 0.17**	− 0.11	− 0.16*	− 0.02	0.14*	0.07	− 0.06	− 0.28**	0.25**	0.23**	(0.89)						
18	PO Fit (t1)	− 0.13	− 0.01	0.06	− 0.14*	− 0.07	− 0.02	− 0.32**	− 0.38**	− 0.41**	0.17*	0.14*	0.18**	− 0.09	− 0.24**	0.25**	0.41**	0.35**	(0.76)					
19	Exhaustion (t1)	0.11	− 0.07	0.01	0.02	− 0.08	0.06	0.54**	0.44**	0.57**	− 0.30**	− 0.36**	− 0.38**	0.29**	0.30**	− 0.24**	− 0.33**	− 0.21**	− 0.34**	(0.84)				
20	Exhaustion (t2)	0.08	− 0.06	0.01	0.01	− 0.01	− 0.01	0.39**	0.56**	0.56**	− 0.45**	− 0.27**	− 0.42**	0.24**	0.26**	− 0.17*	− 0.21**	− 0.23**	− 0.29**	0.65**	(0.86)			
21	Exhaustion (t12)	0.10	− 0.07	0.01	0.02	− 0.05	0.03	0.51**	0.55**	0.62**	− 0.42**	− 0.34**	− 0.44**	0.29**	0.31**	− 0.22**	− 0.29**	− 0.25**	− 0.35**	0.90**	0.92**	(0.90)		
22	ITLorg (t2)	0.14*	− 0.10	− 0.15*	< 0.01	0.01	0.02	0.19**	0.32**	0.31**	− 0.16*	− 0.10	− 0.15*	0.12	0.22**	− 0.02	− 0.16*	− 0.15*	− 0.21**	0.35**	0.41**	0.42**	(0.78)	
23	ITLprof (t2)	0.04	− 0.04	0.11	0.05	− 0.08	< 0.01	0.25**	0.42**	0.39**	− 0.31**	− 0.25**	− 0.33**	0.18**	0.19**	− 0.14*	− 0.24**	− 0.14*	− 0.31**	0.38**	0.53**	0.51**	0.27**	(0.76)
	*M*	0.80	0.28	41.12	0.12	0.09	34.58	3.13	3.06	3.10	3.05	3.14	3.09	3.10	2.66	3.19	3.61	3.91	5.85	3.19	3.36	3.27	1.41	1.39
	*SD*	0.40	0.45	10.97	0.32	0.28	6.79	0.92	0.99	0.82	0.67	0.64	0.56	0.67	0.80	0.60	0.75	0.71	0.84	1.26	1.41	1.21	0.71	0.69

*Note. N* = 222. t = time of assessment (t1 = start, t2 = 12 months later); t12 = mean between t1 and t2; Qualification (0 = ‘Nursing assistant’, 1 = ‘Registered nurse’); Leadership Position (0 = ‘No’, 1 = ‘Yes’); Sex (0 = ‘Female’, 1 = ‘Male’); Contract (0 = ‘Permanent’, 1 = ‘Fixed-term’); PO = person-organisation; ITLorg = intention to leave the organisation; ITLprof = intention to leave the profession. Internal consistency estimates (Cronbach’s α) are in parentheses on the diagonal

* *p* ≤ .05, ** *p* ≤ .01

### Hypotheses testing

#### Job demands and resources as predictors of exhaustion

The results of testing Hypotheses 1 and 2 regarding the relationships between job demands, resources, and exhaustion are displayed in Table 2. After adjusting for depressive mood, we found that time pressure and social conflicts significantly positively predicted exhaustion (Model 2), which aligns with Hypothesis1. However, we note that the effect for social conflicts was no longer significant when adjusting for the four resources in Model 3 in the regression model, because social conflicts and resources (i.e., supervisor support, task identity, and PO fit) shared a common variance, and were also related to exhaustion.

**Table 2:** Results of Hierarchical Regression Analyses for Relationship Between Job Demands, Job Resources, and Exhaustion (Hypotheses 1 and 2)

		Exhaustion^a^							
		M1		M2		M3		M4	
		β		β		β		β	
*Controls*									
	Depressive Mood^a^	0.62	***	0.56	***	0.51	***	0.50	***
*Job Demands*^b^ (Hypothesis 3)									
	Time Pressure (TP)			0.14	*	0.14	**	0.11	*
	Social Conflicts (SC)			0.12	*	0.08		0.10	
*Resources* ^b^									
	Job Control (JC)					− 0.10		− 0.11	*
	Supervisor Support (SS)					− 0.09		− 0.08	
	Task Identity (TI)					− 0.06		− 0.06	
	PO Fit (POF)					− 0.03		− 0.04	
*Demands x Resources*^b^ (Hypothesis 4)									
	TP x JC							− 0.04	
	TP x SS							− 0.06	
	TP x TI							0.14	*
	TP x POF							− 0.11	
	SC x JC							− 0.09	
	SC x SS							− 0.08	
	SC x TI							− 0.09	
	SC x POF							0.06	
	*F*	138.82	***	53.18	***	25.05	***	13.81	***
	*R*² (*p* for Δ*F*)	0.38	***	0.42	***	0.43	*	0.47	**

*Note*. *N* = 222. M1…M4 = Model 1…Model 4; β = standardized regression weight; *F* = *F*-test for model fit against a null model; *R*² = explained variance in exhaustion. Variables were *z*-standardised before calculating the interaction terms

^a^ assessed at t12 (mean between t1 and t2). ^b^ assessed at t1

* *p* ≤ .05, ** *p* ≤ .01, *** *p* ≤ .001

In Hypothesis 2, we assumed that increasing resources would reduce the positive relationship between job demands and exhaustion. However, we found only weak evidence for this assumption (see Table 2, Model 4). From the eight potential interaction terms tested in parallel, only one was significant. However, the pattern of effect was contrary to our assumptions. The slope between time pressure and exhaustion was significantly positive for nurses who reported high task identity (+ 1 *SD*; *B* = 0.31, *t* = 3.22, *p* = .002) and insignificant for nurses who reported low task identity (-1 *SD*; *B* = -0.03, *t* = -0.33, *p* = .744). Therefore, Hypothesis 2 is rejected. However, we noted that our approach of testing the interactions might be conservative when considering the many predictors in a regression model with this sample size. Therefore, we conducted sensitivity analyses and tested all eight interactions (two job demands x four resources) in separate regression models (adjusted for depressive mood and all other work characteristics that were not considered to be independent or moderating variables). We found the following three significant interaction effects to predict exhaustion: time pressure x job control (β_Interaction_ = -0.11, *t* = -2.61, *p* = .01; *B*_*low job control (−1SD)*_ = 0.23, *t* = 3.71, *p* < .001; *B*_*high job control (+1SD)*_ = 0.02, *t* = 0.23, *p* = .818), time pressure x supervisor support (β_Interaction_ = -0.12, *t* = -2.17, *p* = .031; *B*_*low support (−1SD)*_ = 0.27, *t* = 3.41, *p* < .001; *B*_*high support (+1SD)*_ = 0.02, *t* = 0.26, *p* = .794), and social conflicts x supervisor support (β_Interaction_ = -0.10, *t* = -2.11, *p* = .036; *B*_*low support (−1SD)*_ = 0.19, *t* = 2.54, *p* = .012; *B*_*high support (+1SD)*_ = -0.01, *t* = -0.18, *p* = .854). The patterns of slopes between job demands and exhaustion for low and high values of the moderators aligned with our assumptions in Hypothesis 2. Note that the two interaction effects of time pressure x PO fit (β_Interaction_ = -0.10, *t* = -1.90, *p* = .058) and social conflicts x job control (β_Interaction_ = -0.09, *t* = -1.76, *p* = .079) only marginally failed to reach significance (patterns of slopes aligned with Hypothesis 2). Essentially, the results of testing Hypothesis 2 conflicted and depended on the analytical approach used.

#### Exhaustion as a predictor of leaving intentions

The results for testing Hypotheses 3 and 5 on relationships between exhaustion and leaving intentions are displayed in Table 3. We tested six consecutive models. In Model 1, we adjusted for depressive mood, in Model 2 for job demands, in Model 3 for resources, and in Model 4 for the interaction terms between job demands and resources. We added the direct effect of exhaustion in Model 5 and the potential interaction terms with age and health in Model 6.

**Table 3:** Results of Hierarchical Regression Analyses for Exhaustion as a Predictor of Leaving Intentions (Hypotheses 3 and 5)

		ITLorg^a^													ITLprof^a^											
		M1		M2		M3		M4		M5		M6			M1		M2		M3		M4		M5		M6	
		β		β		β		β		β		β			β		β		β		β		β		β	
*Controls* ^b^																										
	Depressive Mood	0.31	***	0.26	***	0.23	**	0.23	**	0.06		0.03			0.39	***	0.36	***	0.28	***	0.23	***	0.04		0.04	
*Job Demands* ^c^																										
	Time Pressure (TP)			0.03		0.03		0.03		− 0.01		0.04					0.08		0.09		0.10		0.06		0.04	
	Social Conflicts (SC)			0.14	*	0.10		0.14		0.10		0.08					0.07		0.03		0.04		0.01		− 0.02	
*Resources* ^c^																										
	Job Control (JC)					0.06		0.05		0.09		0.08							− 0.05		− 0.05		− 0.01		0.00	
	Supervisor Support (SS)					− 0.07		− 0.04		− 0.01		− 0.04							0.00		− 0.01		0.02		0.04	
	Task Identity (TI)					− 0.01		− 0.02		0.00		0.05							− 0.08		− 0.10		− 0.08		− 0.08	
	PO Fit (POF)					− 0.08		− 0.05		− 0.03		− 0.04							− 0.14		− 0.13		− 0.11		− 0.11	
*Demands x Resources* ^c^																										
	TP x JC							0.05		0.06		0.03									0.05		0.07		0.05	
	TP x SS							0.03		0.05		0.02									0.06		0.08		0.11	
	TP x TI							0.05		0.00		− 0.04									0.04		− 0.01		− 0.04	
	TP x POF							− 0.16		− 0.12		− 0.09									− 0.17	*	− 0.13		− 0.08	
	SC x JC							− 0.02		0.01		0.03									− 0.08		− 0.05		− 0.05	
	SC x SS							− 0.17	*	− 0.14		− 0.14									− 0.07		− 0.04		− 0.05	
	SC x TI							0.05		0.08		0.07									− 0.17	*	− 0.13		− 0.07	
	SC x POF							− 0.01		− 0.03		− 0.01									0.05		0.03		0.03	
*Strain* (H3a and H3b)^b^																										
	Exhaustion (t12)									0.35	***	0.41	***										0.38	***	0.35	***
*Moderators* (H5a and H5b)																										
	Age^a^											− 0.15	*												0.08	
	Age x Exhaustion											− 0.21	**												− 0.02	
	Health^b^											− 0.01													− 0.09	
	Health x Exhaustion											− 0.05													− 0.20	**
	*F*	22.58	***	9.17	***	4.37	***	2.75	***	3.80	***	3.89	***		40.53	***	14.66	***	7.64	***	4.59	***	6.07	***	5.87	***
	*R*² (*p* for Δ*F*)	0.09	***	0.10		0.10		0.11		0.17	***	0.21	**		0.15	***	0.16		0.17		0.20		0.27	***	0.31	**

*Note. N* = 222. M1…M6 = Model 1…Model 6; ITLorg = intention to leave the organisation; ITLprof = intention to leave the profession; β = standardised regression weight; *F* = F-test for model fit against a null model; *R*² = explained variance in outcome. Variables were *z*-standardised before calculating the interaction terms

^a^ assessed at t2 (12 months after t1), ^b^ assessed at t12 (mean between t1 and t2), ^c^ assessed at t1

* *p* ≤ .05, ** *p* ≤ .01, *** *p* ≤ .001

In line with Hypothesis 3, even after adjusting for several variables in Models 1 through 4, we found exhaustion to be a significant predictor of ITLorg and ITLprof in Model 5, which confirms Hypotheses 3a and 3b. Notably, the significant effect of depressive mood dropped to an insignificant level after considering exhaustion.

**Fig. 2:**
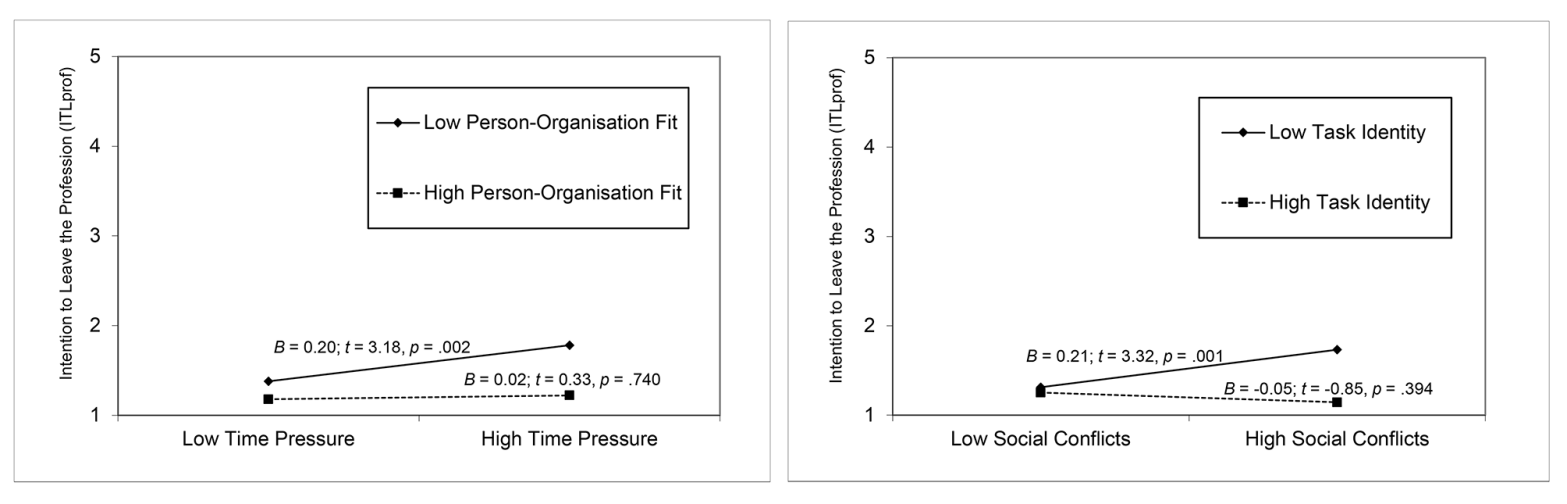
Interaction of Time Pressure and Person-Organisation Fit as Well as Social Conflicts and Task Identity to Predict Professional Leaving Intentions. Note: Mean scores for ITLprof ranging from 1 = ‘Never’ to 5 = ‘Every day’. Estimates were calculated for 1 *SD* below and 1 *SD* above average for time pressure and PO fit

These analyses revealed interaction effects between social conflicts and social support to predict ITLorg and between time pressure and PO fit as well as between social conflicts and task identity to predict ITLprof. Some patterns are displayed in Fig. 2 and suggest that resources can mitigate the positive relationships between job demands and nurses’ leaving intentions. However, effects dropped to an insignificant level in Model 5 when we implemented exhaustion in the regression model, thus providing initial support for its potentially mediating role concerning relationships between work characteristics and leaving intentions.

#### Indirect effects of exhaustion

Results for the potentially indirect effects of exhaustion as a variable that connects job demands and leaving intentions are shown in Table 4. We found relatively robust indirect effects of exhaustion on relationships between time pressure, social conflicts, and both types of leaving intentions when adjusting for depressive mood and the other job demands (Models 1 and 2). However, the indirect effects of exhaustion on relationships between social conflicts, ITLorg, and ITLprof decreased slightly to insignificant levels when further adjusting for all four resources, which might be due to a loss of statistical power for detecting indirect effects. Therefore, we conclude that Hypotheses 4a and 4b are widely supported by our data.

**Table 4:** Results for Testing Indirect Effects of Exhaustion (Hypothesis 4)

			Exhaustion^a^ as a Mediating Variable Between the Independent Variable and Dependent Variable							
			Total Effect			Direct Effect			Indirect Effect	
IV^b^	DV^c^	Model	PE	95% CI		PE	95% CI		PE	95% CI
Time Pressure	ITLorg	1	0.054	[0.076; 0.184]		− 0.004	[-0.131; 0.123]		0.058	[0.020; 0.112]
		2	0.033	[-0.099; 0.164]		− 0.016	[-0.144; 0.111]		0.049	[0.015; 0.102]
		3	0.085	[-0.042; 0.211]		0.029	[-0.093; 0.151]		0.056	[0.015; 0.121]
	ITLprof	1	0.093	[-0.032; 0.218]		0.028	[-0.092; 0.148]		0.065	[0.024; 0.135]
		2	0.082	[-0.045; 0.209]		0.026	[-0.095; 0.147]		0.056	[0.017; 0.120]
		3	0.030	[-0.102; 0.162]		− 0.021	[-0.151; 0.108]		0.051	[0.015; 0.110]
Social Conflicts	ITLorg	1	0.140	[0.009; 0.271]		0.089	[-0.038; 0.217]		0.051	[0.014; 0.103]
		2	0.135	[0.002; 268]		0.091	[-0.038; 0.220]		0.043	[0.010; 0.093]
		3	0.103	[-0.037; 0.243]		0.072	[-0.063; 0.207]		0.031	[-0.004; 0.083]
	ITLprof	1	0.082	[-0.046; 0.209]		0.021	[-0.100; 0.142]		0.060	[0.017; 0.128]
		2	0.068	[-0.061; 0.197]		0.018	[-0.105; 0.140]		0.050	[0.010; 0.117]
		3	0.034	[-0.100; 0.167]		0.000	[-0.127; 0.127]		0.033	[-0.005; 0.094]

*Note*. *N* = 222. PE = point estimate; 95% CI = lower and upper limits of the 95% confidence interval of the point estimates; IV = independent variable; DV = dependent variable; ITLorg = intention to leave the organisation; ITLprof = intention to leave the profession. *Z*-standardised variables were used. Indirect effects were tested using 5,000 bias-corrected bootstrap samples

Model 1 = adjusted for depressive mood; Model 2 = adjusted for depressive mood + other job demands; Model 3 = adjusted for depressive mood + other job demands + job resources (job control, supervisor support, task identity, and PO fit)

^a^ assessed at t12 (mean between t1 and t2). ^b^ assessed at t1. ^c^ assessed at t2

95% CIs excluding zero are significant at *p* < .05

However, a limitation of these results might be to ignore the potentially moderating effects of resources, as outlined previously. Thus, we conducted supplementary analyses and examined moderated mediation models for all combinations of job demands x resources → exhaustion → leaving intention (PROCESS plug-in from Hayes [62]; Model 3; adjusted for depressive mood and other job demands; see Table 5). We found moderated mediations for job control, supervisor support, and PO fit for the effects of time pressure on exhaustion, ITLorg, and ITLprof; additionally, we found moderated mediations for supervisor support on the effects of social conflicts on exhaustion, ITLorg, and ITLprof (the index of the moderated mediations was significant). Inspecting the interaction patterns in more detail, we found that for these relationships, the indirect effects of exhaustion were significant at low levels of the respective resources but not at high levels.

**Table 5:** Results for Testing Moderated Mediations with Exhaustion as the Mediator

						Indirect Effects of Exhaustion^a^				
			Index of Moderated Mediation			Moderator at -1 *SD*			Moderator at + 1 *SD*	
IV^b^	DV^c^	Moderator^b^	PE	95% CI		PE	95% CI		PE	95% CI
Time Pressure										
	ITLorg	Job Control	− 0.042	[-0.088; − 0.014]		0.093	[0.044; 0.166]		0.008	[-0.044; 0.063]
		Supervisor Support	− 0.040	[-0.090; − 0.008]		0.088	[0.039; 0.179]		0.009	[-0.034; 0.065]
		Task Identity	0.020	[-0.013; 0.069]		0.021	[-0.029; 0.086]		0.061	[0.017; 0.136]
		PO Fit	− 0.031	[-0.078; − 0.002]		0.079	[0.032; 0.157]		0.018	[-0.030; 0.071]
	ITLprof	Job Control	− 0.045	[-0.096; − 0.015]		0.100	[0.044; 0.186]		0.009	[-0.042; 0.070]
		Supervisor Support	− 0.048	[-0.108; − 0.012]		0.106	[0.043; 0.213]		0.010	[-0.042; 0.073]
		Task Identity	0.023	[-0.014; 0.072]		0.024	[-0.026; 0.106]		0.070	[0.020; 0.153]
		PO Fit	− 0.035	[-0.084; − 0.002]		0.089	[0.036; 0.179]		0.020	[-0.028; 0.088]
Social Conflicts										
	ITLorg	Job Control	− 0.033	[-0.081; 0.006]		0.083	[0.028; 0.159]		0.016	[-0.041; 0.078]
		Supervisor Support	− 0.030	[-0.076; − 0.005]		0.062	[0.019; 0.138]		0.002	[-0.045; 0.053]
		Task Identity	− 0.024	[-0.066; 0.012]		0.060	[0.011; 0.130]		0.012	[-0.040; 0.074]
		PO Fit	− 0.013	[-0.058; 0.022]		0.051	[0.003; 0.122]		0.025	[-0.024; 0.091]
	ITLprof	Job Control	− 0.036	[-0.089; 0.002]		0.089	[0.028; 0.177]		0.017	[-0.040; 0.082]
		Supervisor Support	− 0.037	[-0.094; − 0.008]		0.076	[0.021; 0.170]		0.002	[-0.051; 0.071]
		Task Identity	− 0.025	[-0.074; 0.010]		0.064	[0.010; 0.143]		0.013	[-0.043; 0.076]
		PO Fit	− 0.015	[-0.064; 0.022]		0.058	[0.003; 0.135]		0.029	[-0.027; 0.101]

*Note. N* = 222. PE = point estimate; 95% CI = lower and upper limits of the 95% confidence interval of the point estimates; IV = independent variable; DV = dependent variable; ITLorg = intention to leave the organisation, ITLprof = intention to leave the profession. Indirect effects were tested using 5000 bias-corrected bootstrap samples. Model adjusted for depressive mood and other job demands. *Z*-standardised variables were used

^a^ assessed at t12 (mean between t1 and t2). ^b^ assessed at t1. ^c^ assessed at t2

95% CIs excluding zero are significant at *p* < .05

In line with Hypothesis 5a, the interaction term of age x exhaustion became significant in Model 6 for ITLorg but not for ITLprof (see Table 3). The slope between exhaustion and ITLorg was significantly positive for younger nurses (-1 *SD*; *B* = 0.44, *t* = 5.72, *p* < .001) but not for older nurses (+ 1 *SD*; *B* = 0.14, *t* = 1.77, *p* = .078).

**Fig. 3:**
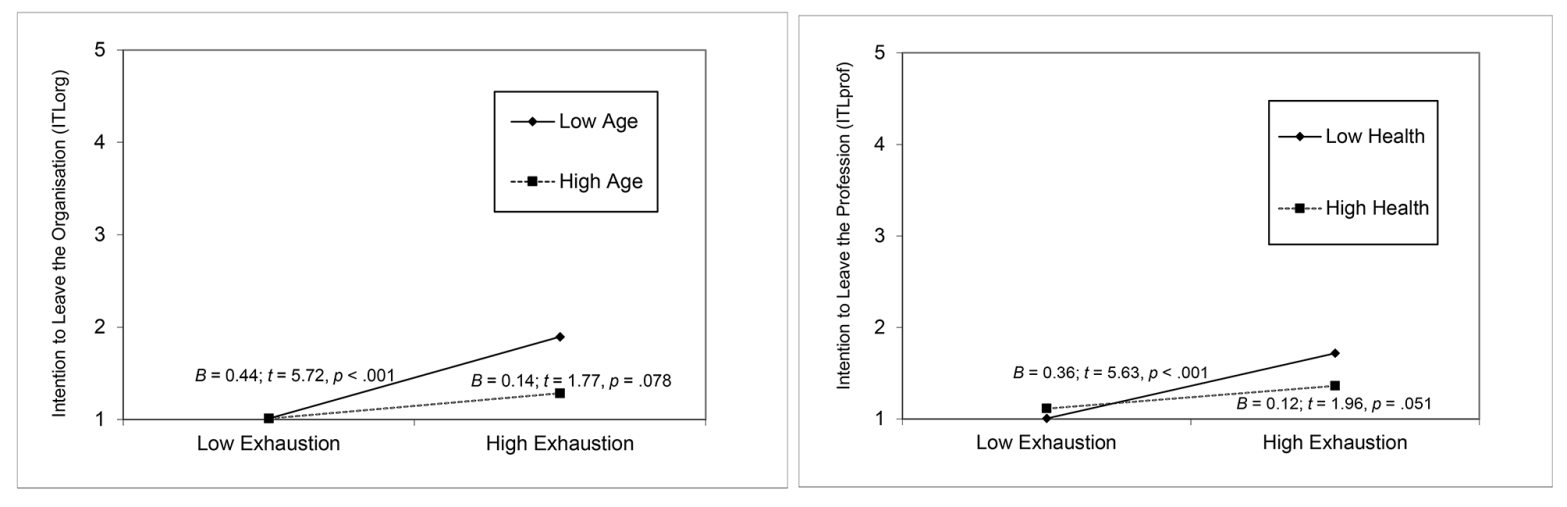
Interaction of Exhaustion and Age or Health to Predict Organisational and Professional Leaving Intentions. Note: Mean scores of ITLorg and ITLprof ranging from 1 = ‘Never’ to 5 = ‘Every day’. Estimates were calculated for 1 SD below and 1 SD above average for exhaustion, age, and health

Moreover, and in line with Hypothesis 5b, the interaction term of health x exhaustion became significant in Model 6 for ITLprof but not for ITLorg (see Table 3). Both interaction plots are illustrated in Fig. 3. The slope between exhaustion and ITLprof achieved a stronger positive result for nurses who had lower self-reported health (-1 *SD*; *B* = 0.36, *t* = 5.63, *p* < .001) than for nurses who had higher self-reported health (+ 1 *SD*; *B* = 0.12, *t* = 1.96, *p* = .051).

## Discussion

Our aim was to uncover hidden mechanisms that predict the organisational and professional leaving intentions of nurses in geriatric care to prevent staff shortages and to preserve nurses’ health and well-being in light of demographic change. The increasing share of older employees within the profession highlights the urgency of maintaining healthy nurses at every age. However, the majority of turnover models do not explicitly reflect the problem of an ageing, health-deprived workforce. Therefore, we chose the JD-R model as an established theoretical framework to integrate research insights on burnout and turnover intention, with a particular focus on age and health. To test the model, we included job demands and resources that are essential in the nursing profession as antecedents of exhaustion and leaving intentions. We also explicitly distinguished between the intention to leave the organisation and the profession.

The present study further adds to the literature by examining the mechanisms that precede nurses’ leaving intentions over a 12-month period. We know from previous research that leaving intentions emerge gradually, often in an interval of six to 12 months [4, 22]. Time pressure and social conflicts are positively associated with exhaustion in our study, which confirms Hypothesis 1 and the postulated demands-strain path of the JD-R model [16, 18]. As time pressure is a typical demand in nursing, researchers sometimes forget that demands can also come from the social environment. Additionally, conflicts with co-workers imply more than just the lack of support and are a serious threat to nurses’ well-being and mental health [63]. Negative workplace interactions, including undermining or bullying among co-workers, seems to have increased in recent years [16, 63]. Both demands are likely related: time pressure leads to additional stress, which leads to additional errors, that must be corrected by another nurse, who becomes angry about the additional work. Moreover, stress causes irritation, which again increases workplace tension.

Initially, we found only weak evidence for Hypothesis 2, which proposed the interaction effects of job demands and resources on exhaustion. Only the interaction term between time pressure and task identity was significant. This may seem counterintuitive, since the slope of high task identity formed in an unexpected direction. However, it may be equally or even more stressful to appropriately perform complete tasks in nursing, such as washing, dressing, and feeding a patient, when time is significantly limited. Although the job characteristics model [23] considers task identity to be a positive characteristic of the job, it may be affected by the stress from time pressure. Additionally, the effect is only marginal and should not be overrated. When we examined the assumed interaction effects of demands and resources individually, we found that the absence of resources increased the positive relationships between job demands and exhaustion, which means that exhaustion is higher under significant time pressure when job control is low. Task identity buffers against social conflicts because tasks are not split between nurses—one nurse is fully responsible for her own mistakes and thus cannot blame a co-worker. In our study, PO fit was important in predicting exhaustion and motivation for remaining in the organisation or profession, which has been investigated in only a few studies so far [12, 29]. Consequently, in line with the core assumptions of the JD-R model, all interaction effects in our study are typical examples of the buffering role of resources and demonstrate that exhaustion is more likely to develop due to the absence of resources when job demands are high. Our third hypothesis, which claims that exhaustion is positively associated with the intention to leave the organisation and the profession, is fully supported by our data. Nurses who felt emotionally drained and burned out from their work were more likely to consider quitting one year later; this finding is supported by previous research [4, 7], although these findings have typically been in cross-sectional studies. Faced with adverse working conditions, understaffing, or a lack of support from supervisors and co-workers, nurses are likely to begin actively seeking alternatives and may eventually leave. This is detrimental not only for the geriatric care institution but also for the entire sector, which is already demonstrating a shortage of qualified personnel in several countries [2]. As we assumed in Hypothesis 4, exhaustion is a linking variable between job demands and leaving intentions, even when controlling for depression. This aligns with the JD-R model and once again emphasises the central role of exhaustion as a precursor of nurses’ intention to leave the job or the profession. Another contribution of our study to existing turnover research is our finding of the moderating effects of age and health in the relationship of exhaustion on different leaving intentions. Confirming our fifth hypothesis, the positive relationship between exhaustion and intention to leave the organisation was stronger for younger nurses whereas poor self-rated health increased the positive relationship between exhaustion and intention to leave the profession. Although prior studies have identified potential explanations for these effects [4, 35], we are unaware of any study that explicitly examines these moderating effects in which exhaustion is an antecedent with diverse effects for the different leaving intentions. Young employees may be less committed to their employers, and they may be less settled in their job as well as their private lives, and may generally be more open to changes, although individual differences apply [64]. Older nurses, conversely, have typically earned higher positions and invested in their careers; thus, they may be hesitant to start again in another geriatric care institution. However, nurses who feel exhausted are more likely to leave the nursing profession if they rate their health as poor. For instance, musculoskeletal diseases are a major issue in this professional group [65] and a primary reason for premature departure from the nursing profession [66]. The finding aligns with results from several studies regarding entries to early retirement [36].

The moderating effects of age and health on exhaustion and intention to leave are dependent on whether the intention is to leave the job or the profession. Thus, it is important to distinguish between the two concepts. There are essential practical reasons: whether a nurse merely moves to another care organisation or leaves nursing prematurely affects not only the individual nurse but also the increasing staff shortages. Therefore, both types of intentions should explicitly appear in the model.

## Limitations

Although many of our findings align with our expectations and past research, we believe that readers should note some limitations. Although more than 50 geriatric care institutions joined the study, 190 denied participation. Thus, results might be affected by a possible self-selection bias. The prospective design of the study offers several benefits in contrast to widely used cross-sectional studies; however, it does not allow for causal conclusions from the data. The sample was quite homogenous, since all participants came from geriatric care institutions in an enclosed region in Germany. Transferring results to other nations or sectors (such as hospitals) should be done cautiously. Furthermore, as previously stated, the present study may lack statistical power for finding small-sized effects.

Since the beginning of turnover research, scholars have debated whether the intention to leave should be on the same level as actual leaving behaviour [13], although recent meta-analyses have found that leaving intentions are one of the strongest predictors of leaving behaviour [12, 67]. Nurses who intend to leave may remain at their current jobs due to scarce alternatives on the local job market. Nevertheless, individual circumstances may force some to leave the profession or take extended absences from the job. Since all of our data in this study were derived from questionnaires, our results could have been influenced by common method bias [46], although we did not find any statistical evidence to this end.

As we conducted a prospective study over a 12-month period, we used the mean value of exhaustion between the start and end of the study as the predictor for leaving intentions at t2 as proposed by Kim and Beehr [51, 52]. This approach is intended to provide a more accurate value for a highly changeable variable, however, we do not know the true value of exhaustion between measurements.

## Practical implications

This might offer care organisations a theoretical basis for preventive measures to maintain the health and work ability of their employees. The insights from this work complement the model with nursing- and ageing-specific aspects and perspectives. Our study underscores that a healthy work design can prevent nurses from leaving the organisation or the profession prematurely. Care organisations must limit job demands and increase resources, such as offering nurses control and creating complete tasks (i.e., including task organisation, task execution, and task feedback). A decrease in exhaustion due to job demands, such as time pressure, could be solved by hiring additional staff, which in the long term can only be achieved by increasing appreciation for and creating a better image of nursing in society; additionally, increasing wages could bolster interest, although many studies have found that salary does not predict turnover [12, 67]. Moreover, our study suggests that organisations should focus on social issues in the workplace. This is especially valid for those in leadership because a supportive supervisor can buffer the negative effects of adverse working conditions or a poisonous team climate [68]. Trainings can improve leadership skills and communication between nurses, which could minimize misunderstandings and tensions [69]. A buffering role of PO fit was found in this study, which is relevant for the personnel selection process [70]. PO fit means that a person and the organisation supplement each other in certain characteristics. An appropriate fit is, for example, when an organisation fulfils the needs, desires, or motives of their members or when a person is equipped with skills and qualifications that meet the requirements of the organisation, which is the principle used in personnel assessment. Although the number of applicants is critically low in many countries and regions, increasing personnel selection effort will save organisations time and expenses in the long run by, for instance, implementing realistic job previews [71]. Finally, organisations must focus on age and health. Early departures from the organisation may be prevented by raising nurses’ commitment to care institutions by, for example, by supporting professional development, allowing nurses to be in charge, and ensuring that nurses feel appreciated. Nurses who have poor health may desire to leave the profession prematurely; this could be avoided through a combination of preventive measures and medical check-ups in which early signs of health impairments are detected and treated or cured. Finally, individual nurses can increase their personal resources to implement the buffering effects, especially when demands are high.

## Conclusion

Nurses’ level of exhaustion predicts their professional and organisational leaving intentions; age and health indicate specific moderating effects. We identify time pressure and social conflicts as job demands; supervisor support, PO fit, job control, and task identity are the resources that drive these relationships. Therefore, redesigning work as well as improving support and the workplace social climate are promising approaches to improve nurses’ well-being and retention.

## Data Availability

The datasets used and analysed during the current study are available from the corresponding author on reasonable request.

## List of abbreviations

t1: Time 1
t2: Time 2
JD-R model: Job demands-resources model
PO fit: Person-organisation fit
NEXT study: Nurses’ early exit study
RN4CAST study: Registered nurse forecasting study
SALSA: Salutogenetic Subjective Work Analysis
MBI: Maslach Burnout Inventory
ITL: intention to leave
ITLorg: intention to leave the organisation
ITLprof: intention to leave the profession
ICC: intra-class correlations
CFA: confirmatory factor analysis
RMSEA: root mean square error of approximation
CFI: comparative fit index
AIC: Akaike information criterion
PE: point estimate
CI: confidence interval
IV: independent variable
DV: dependent variable
